# In‐hospital recurrent stroke in ipsilateral carotid web patients undergoing thrombectomy

**DOI:** 10.1002/acn3.52161

**Published:** 2024-08-30

**Authors:** Farhan Khan, Narendra Kala, Kelvin Chang, Liqi Shu, Eric D. Goldstein, Radmehr Torabi, Krisztina Moldovan, Mahesh Jayaraman, Nahid Mohammadzadeh, Karen Furie, Shadi Yaghi

**Affiliations:** ^1^ Department of Neurology Brown University Providence Rhode Island USA; ^2^ Department of Neurosurgery Brown University Providence Rhode Island USA; ^3^ Department of Radiology Brown University Providence Rhode Island USA

## Abstract

**Objective:**

Carotid artery web is a possible cause of ischemic stroke, especially in young patients who lack conventional risk factors. The immediate and long‐term outcomes are not well studied. We aimed to determine the association between an ipsilateral carotid web and in‐hospital stroke recurrence.

**Methods:**

We analyzed data from adult patients admitted with an acute anterior circulation large vessel occlusion at a Comprehensive Stroke Center between July 2015 and March 2023. The primary outcome was in‐hospital stroke recurrence and secondary outcome was in‐hospital recurrent LVO. Multivariable logistic regression was performed to examine the association between ipsilateral carotid web and recurrent ischemic stroke and recurrent LVO.

**Results:**

Of the 1463 patients with anterior circulation large vessel occlusion, 27 (1.8%) had an ipsilateral carotid artery web. Patients with carotid web were younger (median age (IQR), 60 years (53–67 years) versus 74 years (62–84 years), *P* < 0.01) and less likely to be Caucasian (60% vs. 80%, p = 0.014). Of the 27 patients with carotid web, 18 (70%) had no identifiable competing stroke mechanism. When compared to patients without ipsilateral carotid web, those with an ipsilateral carotid web had a higher risk of recurrent ischemic stroke (adjusted RR: 4.38, 95% CI: 1.38–13.85) and recurrent ipsilateral large vessel occlusion (adjusted RR: 4.49, 95% CI: 1.41–14.21).

**Interpretation:**

Carotid webs are an under recognized cause of acute large vessel occlusion and are associated with higher risk of early recurrence. Studies are needed to validate our findings and test early revascularization strategies in patients with symptomatic carotid artery webs.

## Introduction

Carotid artery web (CW) is a shelf like linear filling defect located along the posterior wall of the proximal internal carotid artery, considered as a potential intimal variant of fibromuscular dysplasia.^1^, ^2^, ^3^, ^4^ CW disrupts blood flow by projecting into the lumen of internal carotid artery, causing thrombus formation and resulting in ischemic stroke via thromboembolism.^1^ Several observational studies have suggested that CWs are more prevalent in young patients with cryptogenic strokes, with rates ranging from 9.4% to 34%.^5^, ^6^, ^7^, ^8^ One study reported a 17% recurrence rate of ischemic stroke, while another reported a 30% recurrence rate in patients who received the best medical management.^1^, ^2^ For secondary stroke prevention, most patients with ipsilateral CW are treated with antiplatelet therapy, although a subset of patients receives anticoagulation due to an elevated risk of arterial thrombosis following mechanical thrombectomy.^5^, ^6^ Surgical options, including carotid stenting or endarterectomy, are typically reserved for patients who experience recurrent ischemic strokes.^8^, ^9^, ^10^ Given the limited data on early recurrent stroke in patients with an ipsilateral carotid web, acute management strategies are not well studied. Recognizing these knowledge gaps, this study aims to investigate the association between ipsilateral carotid web and in‐hospital stroke recurrence in a population of patients with large vessel occlusion undergoing mechanical thrombectomy.

## Material and Methods

The study was approved by the institutional review board and informed consent was waived given the retrospective nature of this analysis. The data that support the findings of this study are available from the corresponding author upon reasonable request.

### Main analysis

This was an observational retrospective cohort study of patients admitted with a large vessel occlusion (LVO) who underwent mechanical thrombectomy at a comprehensive stroke center between July 2015 and March 2023. We excluded patients who had large vessel occlusion of posterior circulation and cases where vessel imaging (CT angiogram of the neck) was unavailable. The primary outcome was in‐hospital recurrent ischemic stroke, defined as new or worsening neurologic deficits confirmed by the presence of new lesion on brain MRI. The secondary outcome was in‐hospital ipsilateral recurrent LVO, defined as intracranial occlusion of internal carotid artery, first (M1) and second (M2) segment of middle cerebral artery. In patients with CW, we only counted recurrent ischemic strokes and recurrent LVOs ipsilateral to the web. We assessed only recurrent ischemic stroke and large vessel occlusion after the index stroke during the same hospitalization Data collection, including admission, demographics, hospital course, assessment of events and potential outcomes, was performed by reviewing electronic medical records by the approved study personnel. The final adjudication of stroke mechanism is determined by board certified vascular neurologists as per TOAST criteria. The primary and secondary outcomes were adjudicated by the board certified vascular neurologists (F.K. and S.Y.). Any identified carotid webs (S.Y.) and potential outcomes (E.G.) were evaluated by independent reviewers, both of whom were blinded.

### Imaging analysis

The carotid web on CTA was defined as a thin linear filling defect arising from the posterior wall of the proximal internal carotid artery, with a smooth border and without atherosclerosis at the site of the web.^7^, ^8^ The imaging was reviewed in multiplanes (axial, sagittal and coronal) by board certified vascular neurologists (F.K. and N.K.) and neuro‐endovascular surgeons (R.T. and K.M.).

### Statistical analysis

Variables were summarized by means and standard deviations or medians for continuous variables and frequencies/proportions for categorical variables. Differences between groups were evaluated using chi‐squared tests and Fisher's exact tests for categorical variables and parametric (t‐test) for continuous variables as appropriate. Univariate logistic regression was performed to assess the relationship between carotid web and recurrent large vessel occlusion and recurrent ischemic stroke and unadjusted relative risk were reported with 95% confidence intervals (95% CI). Multivariate logistic regression was performed to examine the association between carotid web and primary and secondary outcomes after controlling for age, reporting adjusted relative risk (RR) along with 95% CI. All statistical tests were two sided and were evaluated at a significance level of *P* < 0.05 unless specified otherwise. Statistical analyses were carried out in Stata 18 (StataCorp LLC, College Station, TX, USA).

Based on prior literature, the risk of recurrent LVO and ischemic stroke in patients with anterior circulation LVO is around 0.7–2%.^3^ To detect a 3% difference in recurrent ischemic stroke and LVO in patients with ipsilateral carotid artery web in the hospital with 90% power, a sample size 1200 patients was determined. To account for poor imaging quality, posterior circulation stroke, lack of imaging and missing data, an additional 400 (20%) patients are included with an overall sample size of 1600.

## Results

**Figure 1:**
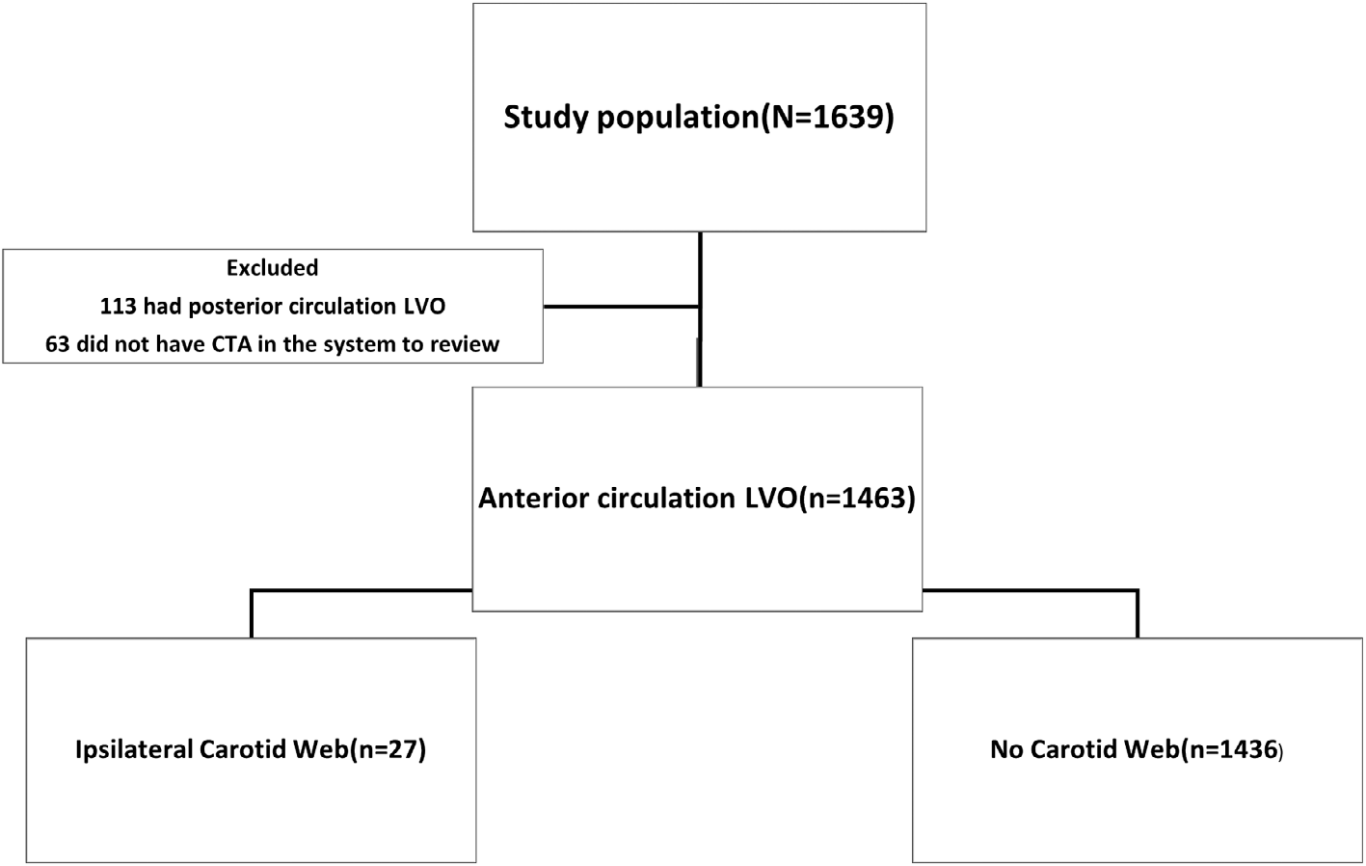
Flow diagram.

Out of 1639 participants diagnosed with large vessel occlusion, 176 were excluded because posterior circulation LVO, and CTA were not available for review (see Fig. 1). Of the remaining 1463, we identified 27 patients (1.85%) who had large vessel occlusion ipsilateral to carotid web. Eight carotid webs (0.55%) were detected on the contralateral side (asymptomatic carotid web) and two patients (0.2%) were found to have bilateral CW. CW were most often detected on the right side (71%).

**Figure 2:**
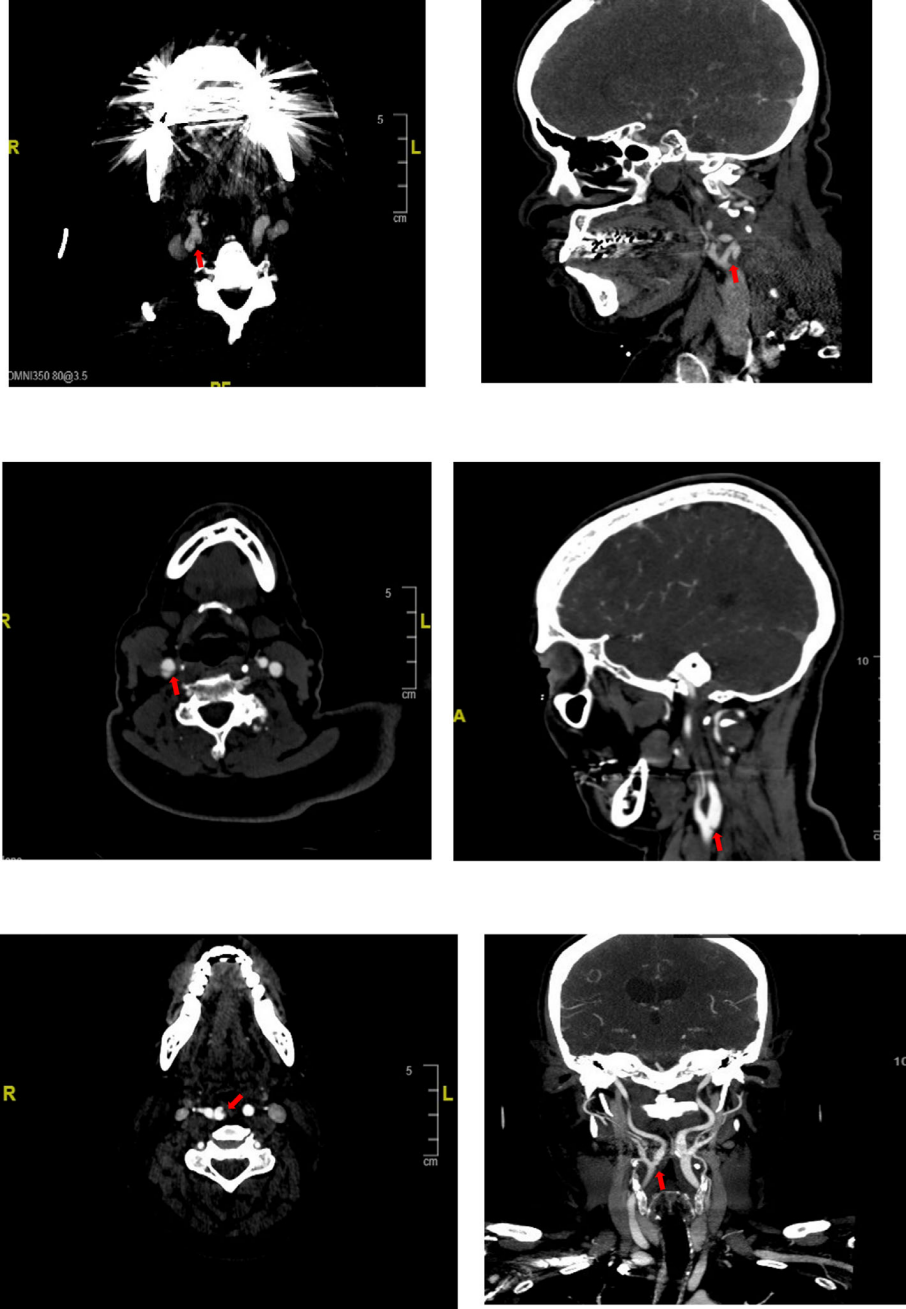
Arrows indicate carotid webs (CW) of patients who developed recurrent ischemic stroke in multiple planes. As patient characteristics are reported in Table 1.

There were 29 recurrent ischemic strokes (~2%) and 28 recurrent LVO (1.9%) in 1463 patients. 11% (3/27) of patients with ipsilateral carotid artery webs experienced recurrent ischemic stroke and LVO. Also, 21 of 24 (87.5%) patients with CW received medical management and 3 (12.5%) patients underwent carotid revascularization (1 carotid endarterectomy and 2 trans‐carotid artery revascularizations). None of these patients developed immediate post‐operative complications. The baseline characteristics and pictures of CW (pre and post intervention) of those who developed recurrent ischemic stroke are presented in Table 1 and Figure 2, respectively.

**Table 1:** Baseline characteristics.

	No web (*n* = 1436)	Carotid web (*n* = 27)	*P*‐value
Age, median (IQR)	74 (62–84)	60 (53–67)	<0.001
Male sex	698 (48.6%)	11 (40.7%)	0.42
Race			
White	1140 (79.4%)	16 (59.3%)	0.014
Black	71 (4.9%)	5 (18.5%)	
Asian	16 (1.1%)	0 (0.0%)	
Other	209 (15%)	6 (11%)	
Hispanic ethnicity	81 (5.6%)	3 (11.1%)	0.48
Hypertension	1087 (75.7%)	15 (55.6%)	0.05
Diabetes mellitus	357 (24.9%)	7 (25.9%)	0.96
Hyperlipidemia	776 (54.0%)	14 (51.9%)	0.95
Atrial fibrillation	600 (41.8%)	8 (29.6%)	0.43
NIHSS, median (IQR)	17 (11–21)	16 (11–21)	0.81
Symptomatic web location			
Right		19 (70.4%)	
Left		8 (29.6%)	
IV thrombolytics	565 (39.4%)	16 (59.3%)	0.037
Medications at baseline			
Aspirin	493 (34.3%)	10 (37.0%)	0.88
Clopidogrel	76 (5.3%)	0 (0.0%)	
Direct oral anticoagulant	144 (10.0%)	0 (0.0%)	0.65
Warfarin	112 (7.8%)	0 (0.0%)	0.86
Location of occlusion			
ICA	267 (18.6%)	5 (18.5%)	0.50
M1	921 (64.1%)	21 (77.8%)	
M2	248 (17.3%)	1 (3.7%)	
Mechanism			
Cardioembolism	606 (45.8%)	6 (23.1%)	<0.01
LAA	206 (15.6%)	2 (7.7%)	
ESUS	380 (28.7%)	18 (69.2%)	
Stroke of other etiologies	129 (9.8%)	0	

Values are expressed as *n* (%). IQR, interquartile range; NIHSS, National Institute of Health Stroke Scale; DOACs, direct oral anticoagulants.

### Univariable analyses

Patients with symptomatic carotid webs were younger when compared to patients without carotid webs (median age (IQR), 60 years (53–67) vs. 74 years (62–84), *P* < 0.01) (Table 1). CWs were more frequently reported in Black patients (4.9% vs. 18.5%, *P* = 0.014) and had a similar baseline risk factor profile, including hypertension, dyslipidemia, atrial fibrillation, and diabetes mellitus, and were similar in both men and women. In 18 out of 27 patients with CW, no other potential cause of stroke was identified, and it was deemed ESUS (67% vs. 29%, *P* < 0.01). Approximately, half of patients (14/27) with CW underwent cardiac monitoring on discharge, and one patient was subsequently found to have atrial fibrillation as compared to patients without CW (17.5% vs. 3.7%, *P* = 0.045). Other variables were statistically non‐significant and shown in Table 2.

**Table 2:** Characteristics of patients with ipsilateral carotid web.

Age	Cardiovascular risk factors	Recurrent stroke in Hospital	TICI post MT	Medications at time of recurrent stroke	Cardiac monitoring on discharge
40s	Smoker	No	3		1
60s	DM, smoker	No	3		0
50s	HTN, smoker	No	3		1
50s	None	No	3		1
70s	HTN, AF, HLD	No	3		0
50s	HTN, smoker	No	3		1
60s	Former smoker	No	2c		1
40s	HLD	No	3		0
50s	HLD, DM	**Yes**	3	Aspirin	1
50s	HLD, DM	No	2c	Aspirin	0
80s	HTN, AF, HLD^1^	No	3		0
40s	HTN, HLD	No	3		0
60s	HTN, current smoker	No	0		1
60s	None	**Yes**	3	Received tpa	1
60s	None	No	0		1
50s	HTN, AF	No	3		0
60s	HLD, ex‐smoker	No	2c		1
50s	DM, HLD	No	2b		0
60s	Former smoker	No	3		1
70s	HTN, AF, HLD, former smoker	No	2c		0
60s	HTN, DM, HLD^1^	No	3		1
50s	HLD, DM	**Yes**	3	Aspirin	1
70s	HTN, AF, former smoker	No	2c		0
40s	HTN, AF, HLD	No	0		0
50s	HTN, HLD, current smoker	No	3		1
70s	HTN, AF, DM, former smoker	No	2c		0
80s	HTN, AF, HLD, former smoker	No	3		0

HTN, hypertension; AF, atrial fibrillation; HLD, hyperlipidemia; DM, diabetes mellitus; TICI, thrombolysis in cerebral infarction; MT, mechanical thrombectomy.

^1^ Indicates smoking status unknown.

#### Association between ipsilateral carotid web and recurrent ischemic stroke

In patients with symptomatic carotid web (CW), an increased incidence of recurrent ischemic stroke was observed for the primary outcome when compared to those without CW in an age‐adjusted model (adjusted RR: 4.38, 95% CI: 1.38–13.85, *P* = 0.012) (Table 2). Furthermore, a similar association was noted in individuals with CW and without an alternative competing mechanism (atrial fibrillation, LV dysfunction, endocarditis) in a similar age‐adjusted model (aRR: 5.64, 95% CI: 1.78–17.89, *P* < 0.001) (Table 3).

**Table 3:** Association between ipsilateral CW and recurrent ischemic stroke and LVO.

	Model 1		Model 2		Model 3		Model 4	
	Unadjusted RR (95% CI)^1^	*P*‐value	Adjusted OR (95% CI)^1^	*P*‐value	Unadjusted OR (95% CI)^2^	*P*‐value	Adjusted OR (95% CI)^2^	*P*‐value
Recurrent ischemic stroke	6.13 (1.98–19.05)	<0.001	4.38 (1.38–13.85)	0.012	8.72 (2.89–26.35)	<0.001	5.64 (1.78–17.89)	<0.001
Recurrent LVO in Hospital	6.37 (2.04–19.84)	<0.001	4.49 (1.41–14.21)	0.01	9.6 (3.71–22.08)	<0.001	5.76 (1.81–18.30)	<0.001

^1^ Models 1 & 2 are unadjusted and adjusted models (age) respectively.

^2^ Models 3 (unadjusted) & 4 (adjusted for age) represents sensitivity analysis for patients with ipsilateral CW and no competing mechanism. LVO indicates large vessel occlusion and RR indicates relative risk.

#### Association between ipsilateral carotid web and recurrent large vessel occlusion (LVO)

For the secondary outcome, 3 out of 27 (11%) developed recurrent large vessel occlusion ipsilateral to carotid web, compared to 20 of 1434 patients (1.75%) during the hospital stay (aRR: 4.49, 95% CI: 1.41–14.21, *P* = 0.01) (Table 2). When restricting the analysis to those with ipsilateral carotid web without an alternative competing mechanism, the risk was increased ~6‐fold for in‐hospital recurrent large vessel occlusion in an age adjusted model (aRR: 5.76, 95% CI: 1.81–18.30, *P* < 0.001) (Table 3).

All recurrent ischemic stroke and large vessel occlusion occurred within 48 h after the index stroke.

## Discussion

Carotid webs are known cause of recurrent ischemic stroke including large vessel occlusion^1^, ^4^, ^5^ Our study shows that patients with carotid web (CW) and large vessel occlusion (LVO) without a clear competing alternative etiology are at increased short‐term risk of in‐hospital recurrent ischemic stroke. The immediate increased risk of recurrent stroke suggests that single antiplatelet therapy may not be sufficient. One study reported an elevated risk of recurrent stroke risk at 2 years among those who were receiving anticoagulation^6^; however, the benefit of immediate dual antiplatelet or anticoagulation therapy remains unclear, particularly in medium to large strokes where there is an elevated risk of hemorrhage.^12^ Several studies have reported decreased rates of long‐term recurrent strokes in individuals undergoing carotid revascularization^10^, ^13^ suggesting that this option is worth considering, especially for those who experience recurrent ischemic strokes ipsilateral to CW. Therefore, the consideration of immediate dual antiplatelet therapy or anticoagulation, without or without endovascular intervention, should be approached cautiously due to a lack of sufficient clinical and safety data.

In patients with CW, the underlying stroke mechanism is attributed to turbulence of blood flow and stagnation in the space posterior to the web, resulting in thromboembolism. This hypothesis finds support in studies demonstrating significant stasis and delayed contrast washout distal to the CW on catheter angiography.^13^, ^14^, ^15^ Additionally, hemodynamic flow patterns suggest increased recirculation times in the area posterior to the web, potentially fostering thrombogenesis through increased platelet deposition, aggregation, and elevated wall shear stress.^16^ In summary, CaW patients have larger regions of hemodynamic parameters associated with clot formation compared to subjects with atherosclerotic lesions or healthy subjects.^9^ This makes it unique and challenging, as majority of the webs do not cause flow limiting carotid stenosis and are often overlooked on imaging.^13^

In our cohort of individuals with large vessel occlusion undergoing thrombectomy, the prevalence of ipsilateral carotid web was 1.85%, which is in the same ballpark as other studies (0.7–2.5%).^6^, ^9^, ^11^ In over two‐thirds of patients (approximately 70%) with ipsilateral carotid web (CW) and index occlusion, an exhaustive workup did not reveal an alternative stroke etiology. As a result, these cases were classified as embolic stroke of undetermined source (ESUS) following diagnostic evaluation. Given that nearly 70% of individuals with ipsilateral CW did not have an alternative identified cause of stroke, it is critical to identify carotid web as an underlying cause.

The immediate increased risk of recurrent stroke suggests the need to optimize antithrombotic treatment regimens and consider early revascularization, akin to the approach for patients with acutely symptomatic carotid stenosis. These findings call for more effective treatment strategies than medical management alone. Growing evidence suggests that stenting and surgical intervention might be effective for these patients. Given the high rates of stroke recurrence among those treated solely with medical management, this area warrants further research.

## Limitations

Our study has several limitations. First, the single center registry on large vessel occlusion could have skewed the overall prevalence and limits generalization. Second, the small number of CWs and fewer outcomes of interest led to wider confidence intervals, and we could not adjust for confounders given small event rate. Third, approximately, 30% of patients had atrial fibrillation in individuals with CW and thus it is challenging to determine the underlying mechanism with certainty. Our findings were even more pronounced in patients without AF, however. Finally, we did not perform long‐term follow‐up on these patients who received either medical or endovascular treatments after the index stroke with CW.

## Conclusion

Patient with CW are at high risk for recurrent LVO and ischemic stroke during their hospital stay. This emphasizes the need for further investigation into additional prevention strategies including optimizing antithrombotic treatment and carotid revascularization.

## Author Contributions

F.K., S.Y., and N. K. contributed to the conception and design of the study. E.D., G.R., K.M., N.M., L.S., and K.C. contributed to the acquisition and analysis of data. M.J. and K.F. contributed to drafting the text.

## Conflict of Interest

None.

## Data Availability

The data that support the findings of this study are available from the corresponding author upon reasonable request.
